# Retrospective Evaluation of Discharge Planning Linked to a Long-Term Care 2.0 Project in a Medical Center

**DOI:** 10.3390/ijerph191610139

**Published:** 2022-08-16

**Authors:** Su-Tsai Huang, Chun-Min Chen, Yu-Yung Su, Shu-Chen Chang

**Affiliations:** 1 Nursing Department, Changhua Christian Hospital, Changhua 500209, Taiwan; 2 Big Data Center, Changhua Christian Hospital, Changhua 500209, Taiwan; 3 Department of Long Term Care, National Quemoy University, Kinmen 892009, Taiwan; 4 College of Nursing and Health Sciences, Dayeh University, Changhua 515006, Taiwan

**Keywords:** long-term care, hospital discharge planning, case-mix system, service use

## Abstract

Background: Although there are several studies on discharge planning and long-term care systems in individual programs, research on the connection between discharge planning and the usage of long-term care is scanty. This study aims to evaluate the nature of the association between discharge planning (DP) and long-term care (LTC) and whether the utilization of LTC services improved after being discharged. Methods: This was a single-center retrospective medical record review study. Secondary data analysis was conducted of DP–LTC participation data between 2018 and 2019. The objectives were to clarify the distinct characteristics of each part of the service to explore the utility rate by overall users and users with willingness and to determine the factors influencing their usage. Medical claims were used to identify inpatients receiving discharge services, and data were matched with LTC system engagement data (*n* = 2155). Backward stepwise regression was used to explore the attributes associated with each type of service use. Results: A total of 94% (2042/2155) of inpatients expressed a perceived need for LTC services, of which 14% (285/2042) were users of LTC services after discharge. When assessed by case-mix system (CMS) and willingness to use services during hospitalization, inpatients had higher rates of service utilization after discharge. Using LTC services was most likely to be associated with obesity, disability, high CMS level, higher education, and women. Conclusion: The study confirms that the utilization of LTC services has improved under the integrated DP–LTC system. The gap between willing and actual users is worth considering. In the assessment stage, special attention should be paid to the service needs of persons with BMI ≥ 27 and disabilities. Future research with a larger sample could comprehensively evaluate the impact of integrated DP services on the use of LTC 2.0 service resources.

## 1. Introduction

Aging has become a major challenge for Taiwanese society. According to a World Bank report, Japan has the largest aging population in the world (28%) [1]. It took only 25 years for Taiwan to reach the aging society standard defined by the World Health Organization (7% to 14%), which is the same rate as that of Japan. It is anticipated that it will exceed 20% in 2026 and thus Taiwan is set to become a super-aged society. This aging rate will be the second highest worldwide. For Taiwan, the transition from an ‘aged society’ to a ‘super-aged society’ will take only eight years, whereas it took 11 years for Japan, 15 years for the US, 29 years for France, and 51 years for the UK [2]. Furthermore, the aging population will present a higher prevalence of functional disabilities, subsequently leading to an increased demand for and burden on long-term care (LTC) [3,4]. Changes in Taiwan’s demographic structure have therefore increased the need for LTC.

In response to society’s concerns about caring for its older population, LTC in Taiwan has been regarded as an important part of policy and has been vigorously promoted. The 10-year LTC plan version 1.0 was launched in 2007 and the reformed LTC plan version 2.0 was launched in 2017 [5]. The payment system for LTC services was further modified in 2018 as part of the LTC 2.0 reforms. The payment system came under universal public-tax-based LTC coverage [5]. As Taiwan is now entering the second decade of LTC development, the current system still needs to be reviewed to ensure that it can face the challenges of an aging population and limited workforce resources [6].

In recent years, the Taiwanese government has promoted diversified resources for LTC services. However, numerous studies have indicated that these services do not deliver as many practical benefits as expected [7]. In Taiwan, according to demographic statistics from 2018 [8], approximately 765,000 people are disabled or live with dementia and 60% of them do not use LTC resources. This could be due to the limitations of LTC 1.0. For example, having services that are only available for certain people, inflexible payment methods (the service hours of the different items were fixed and the payments could not be cross-projected), having a low retention rate of care attendants, etc. Therefore, LTC 1.0 was upgraded to 2.0 in 2017. The service items expanded from eight to 17 and DP was included in the new version [9].

There is evidence from studies that shows that discharge planning can reduce the length of hospital stays, readmission rates, and medical costs as well as improve health care quality and increase patient satisfaction [10,11,12]. Linking long-term care with hospitals can improve the quality of care for chronic patients after returning home [13]. According to the Ministry of Health and Services policy, hospitals established a DP–LTC 2.0 “Dedicated Discharge Planning Team” in July 2018 to promote the connection between discharge preparation and Long-Term Care 2.0 services and assist patients with discharge preparation to transition to Long-Term Care 2.0. DP–LTC care includes logistical arrangements, the education of the patient and family, and coordination between health professionals involved in the transition [14]. It is hoped that through discharge planning services, resources can be integrated to provide a seamless transition from acute care to long-term care. By establishing an integrated discharge plan, the patient and family can transition safely out of the medical facility and return home or transfer to another facility in a timely and safe manner [15]. However, research on the connection between discharge planning and long-term care is scarce [16]. It remains to be seen whether hospital DP and LTC services can be truly integrated after simplifying the delivery flow for access to LTC services.

Considering that little is known about how seamless discharge affects long-term care service use, the present study explored the effects of hospital discharge planning in response to Long-Term Care 2.0 on LTC service utilization patterns and frequency after discharge. Furthermore, the factors associated with the utilization of different services were identified. The utilization gap between the initial intention to use LTC services during hospitalization and actual utilization after discharge was also evaluated, in the hopes that DP–LTC services can achieve the goal of the seamless integration of care and assistance for people in need.

## 2. Methods

### 2.1. Data Sources and Participants

A retrospective medical record review study was conducted in a medical center with 1228 beds in central Taiwan. In this retrospective study, we focused on subjects who received DP services and Multi-dimensional Assessment Instrument (MDAI) assessments at Changhua Christian Hospital (CCH). All eligible patients were included from July 2018 to December 2019, for a total of 2155. The hospital information system provided information on patients who used discharge preparation and received long-term care 2.0 assessments, and who received service items that they applied for through the local health bureau. Data were extracted from electronic databases, including the hospital discharge records of CCH containing International Classification of Diseases 10-Clinical Modification (ICD 10-CM) pathology codes, data on the DP of CCH containing the demographic variables (age, gender, etc.), service use assessments (with and without willingness), and the CMS levels (levels 2–8). Data on the use of LTC resources within three months of discharge was provided through the Department of Health’s LTC Resource Platform.

### 2.2. Multi-Dimensional Assessment Instrument (MDAI) and Long-Term Care Case-Mix System (LTC-CMS)

The government uses the Multi-dimensional Assessment Instrument (MDAI) to measure disability status [17], which evaluates six dimensions:

1. Individual communication skills or short-term memory assessment.
2. Activity of Daily Living (ADLs) and Instrumental Activities of Daily Living (IADLs).
3. Special complex care needs.
4. Home environment and social participation.
5. Emotional and behavioral patterns.
6. Primary caregiver load, work, and support.

The data collected by the MDAI were uploaded to the long-term care service information system established by the government. The information system can automatically determine the long-term care case-mix system (LTC-CMS) need level for each individual and the corresponding payment amount based on the need level [8,18]. In other words, each long-term care service package is based on an individual long-term care need level and the specific assistance needed. Further, the payment amount depends on the degree of the disability and what types of services need to be delivered. The payment system comprises four categories of services including personal and professional care (USD 334–1260/month); transportation (USD 56–80/month); assistive devices and home barrier-free (USD 1333/month); and respite care for caregivers (USD 1078–1617/month) [8].

### 2.3. Measurement

#### 2.3.1. Dependent Variables

The use of LTC services after discharge was derived from LTC resource platform archives. Data from the LTC services contain four types of service use records (personal and professional care services, transportation, assistive devices, home barrier-free services, and respite care).

#### 2.3.2. Independent Variables

Information on the sociodemographic characteristics was collected for the following variables: age at hospital admission (≤69, 70–79, 80+), gender, education (none, primary, junior high, and above), marital status (unmarried or married), income (low or general), living area (urban or rural), disability card holder (yes or no), dementia (yes or no), body mass index (BMI < 18.5, 18.5 ≤ BMI < 24, 24 ≤ BMI < 27, and BMI ≥ 27) [19], and primary caregiver (foreign caregiver, daughter, son, spouse, and other relatives). Information on care needs, as they were evaluated before the patient’s hospital discharge, were classified according to the CMS level (2–8, which is from mild to severe).

#### 2.3.3. Statistical Analyses

**Figure 1:**
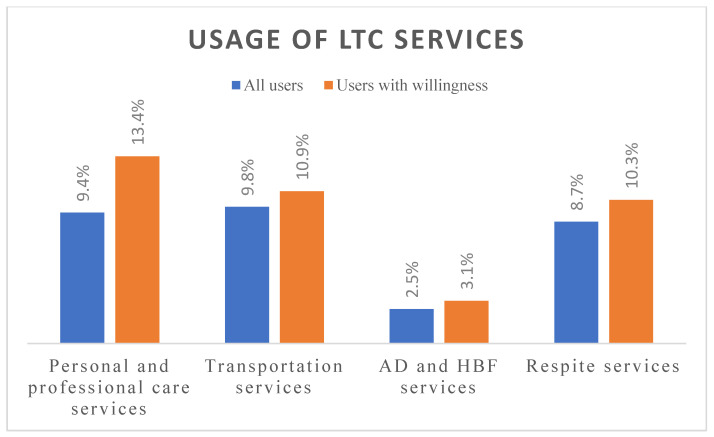
Percentages of “all users” and “users with willingness” by the four LTC services used. (AD: Assistive devices; HBF: home barrier-free).

Descriptive analyses (frequencies and percentages) were performed on the demographic data to characterize the sample (Table 1). Based on the LTC 2.0 payment system, the services were divided into four types. The percentages of “users with willingness” and “all users” by the four types of services used are shown in Figure 1. Further, we examined whether the different levels of CMS provided different services to people with BMI problems or disabilities. We also used stepwise regression analysis to evaluate the effects of the underlying factors on service use. The stepwise selection method is a widely used variable selection method, particularly in the medical field. The backward elimination approach in stepwise selection is often preferred since it takes into account a full model and examines the impact of all candidate variables [20]. To examine the differential impact of the potential factors on the various services used, the regression model was separately performed on each service group. Statistical analyses were performed using SPSS for Windows version 22.0 (IBM Corp., Armonk, NY, USA), and the significance level was set at α = 0.05.

## 3. Results

### 3.1. Descriptive Statistics

The results shown in Table 1 indicate that most of the patients were older than 70 years (79.9%) and married (71.4%). Additionally, 51.5 % of the sample were female, 46.6% were primary-school educated, 93.1% were employed with ‘general income’, 59.9 % lived in rural areas, 30.4% had disabilities, and 6.4% had dementia. More than two in five participants (43.6%) were overweight or had obesity. A total of 63.8% of caregivers of the participants were adult children (son and daughter), 23.6% were spouses, 7.9% were foreign caregivers, and 4.0% were other relatives. The most common CMS level was level 8 (29.5 %), followed by level 7 (23.4 %), level 5 (19.9%), and level 4 (19.3%).

### 3.2. Users by LTC Services

Overall, 94% (2042/2155) of inpatients expressed a perceived need for LTC services, of which 14% (285/2042) were users of LTC services after discharge. Figure 1 displays the percentages of actual users of LTC services (personal and professional care, transportation, assistive devices and home barrier-free, and respite care) after being discharged. The utilization of “personal and professional care” services was 9.4% overall and 13.4% in patients with willingness. “Transportation service” usage was 9.8% overall and 10.9% in those with willingness. “Assistant devices and home barrier-free” service usage was only 2.5% overall and 3.1% in those with willingness. Finally, 8.7% used respite services overall, whereas 10.3% of those with willingness used these services. The results showed that inpatients who were assessed by CMS and willingness to use services during hospitalization had higher service utilization rates after discharge.

### 3.3. Backward Stepwise Regression Model for Factors in Referrals to LTC Services

The factors affecting LTC service use are shown in models A, B, and C (Table 2). Those who were obese (OR = 1.46; 95% CI = 1.01–2.12) were more likely to seek personal or professional care services (in Model A). A strong preference for transportation services was observed among older adults with obesity (OR = 1.81), disability (OR = 1.47), a high education level (OR = 1.51), and a high CMS level (OR = 1.16) (in Model B). Female elderly adults (OR = 1.39) and those with disability cards (OR = 1.57) had strong preferences for respite services, as shown in Model C. As no significant impact factors were found in model D (AD and HBF services), the model is not presented in Table 2.

### 3.4. CMS-Related Factors That Affect Service Use

**Figure 2:**
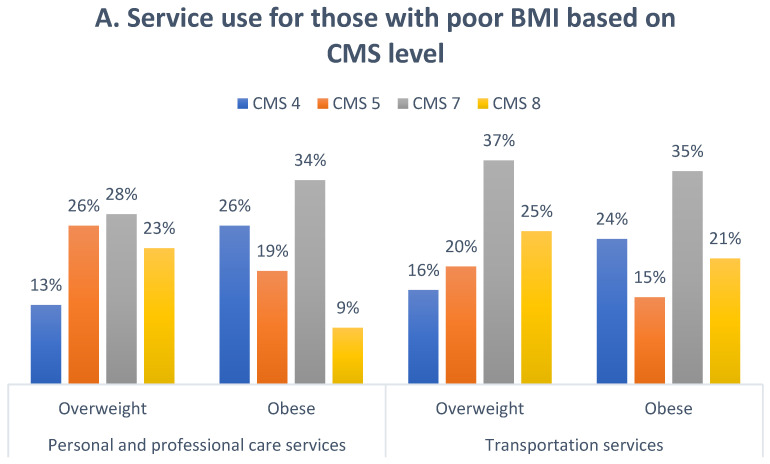

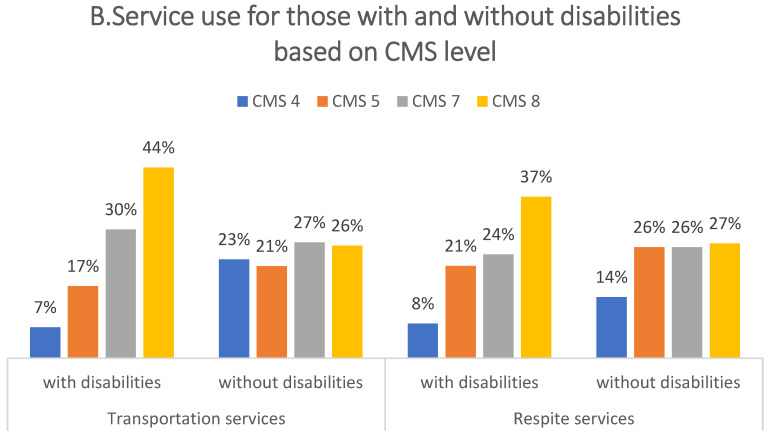
(**A**) Percentages of overweight and obese participants by “personal and professional care” and “transportation” services used based on CMS level. (**B**) Percentages of participants with and without disabilities by “transportation” and “respite” services used based on CMS level.

Figure 2A,B display the percentages of CMS levels for those who were overweight, obese, or with or without disability cards by three LTC service utilizations (personal and professional care, transportation, and respite services). In most types of LTC services, the majority of services used were attributed to CMS levels 7 and 8. This was especially relevant in the case of transportation services, which were used mainly by participants who were overweight (37%) and obese (35%). In addition, people with disability cards used more transportation (44%) and respite (37%) services than those without disability cards. Accordingly, participants with CMS level 7 with a BMI > 24 or CMS level 8 with a disability were more likely to use both in-home care and community support services.

## 4. Discussion

The aim of this study was to evaluate whether a connection between DP and LTC improved the utilization of LTC services after patients were discharged from the hospital. We used a sample from a medical center in central Taiwan to investigate changes in LTC service utilization among patients after their discharge. In summary, those with poor BMI status, disability cards, severe CMS, older age, higher education, and who were female were the best-predicted users of LTC services; however, for each service type, every factor was not equally important. This study found that the utilization rate of LTC services under the DP–LTC integrated system improved slightly. Unexpectedly, the CMS only produced a positive correlation with the use of transportation services. In the assessment stage, special attention should be paid to the service needs of persons with BMI ≥ 27 and disabilities.

To improve the accessibility of LTC services, LTC 2.0 is linked to the hospital’s discharge plan through a seamless integration model to provide uninterrupted in-home care [8]. The ratio of users who were willing to use LTC services was 13.6%, which is higher than that of countries with insurance-covered LTC such as Germany (5.9%) and Japan (7.14%) [21]. However, compared with Yin’s study [22], the transition rate to LTC use in CCH is relatively low (14% vs. 26.9%). Our study highlights a significant disparity between the perceived need for LTC services and actual service use. This gap highlights areas for improvement. It is crucial to find out which links cause the high willingness but low usage problems. There are several mechanisms that can explain the observed association. First, in-hospital long-term care case screening information should be reviewed to reduce the erroneous screening of non-compliant cases. Yin’s study [22] also confirmed that the reason for the low utilization of long-term care services after discharge is that long-term care case screening information is not practical. Second, the suitability of a needs assessment within 24 h of the acute phase of admission needs to be reviewed. This can be explained by the fact that the included cases did not fully meet the long-term care screening criteria; the patients were stable after the acute phase so there was no willingness to use long-term care services after discharge. Third, informal care provided by a spouse can reduce the need for long-term care [23] given that 71% (1532/2155) were married and 32.3% (495/1532) of primary caregivers were spouses. Compared to the elderly who live with adult children, it is also possible that the elderly who live with their spouses are less likely to use paid services [24]. Another possibility is that community care may be underestimated because the records of service usage do not come from the national integrated information platform [6].

Barriers to using long-term care services are related to low education and low public awareness [25,26,27]. Our research confirms that higher education levels are associated with greater transportation usage. About 28% of elderly people in our study were illiterate and 46.6% graduated from elementary school. It is often challenging for elderly people with low education levels to obtain accurate health information, resulting in barriers to obtaining LTC and healthcare. Yin’s study [22] also pointed out that the low utilization rate of long-term care services after discharge could be due to the misunderstanding of patients and their families about the services provided by long-term care. Promoting LTC services during hospitalization (by creating promotional videos, providing guidance leaflets, and organizing lectures) is recommended. Consequently, LTC services and education must explore ways to improve communication with low-education LTC recipients and their primary caregivers while meeting their needs and increasing the utilization of LTC services.

Notably, early CMS assessments could have improved patient and caregiver awareness of long-term service resources and increased utilization, but only transportation service use was positively associated with CMS level. Several mechanisms could explain the observed associations. First, people with high levels of CMS could have high formal medical care needs, thereby eliminating the need for LTC services. Health conditions associated with disabilities could result in poor health and extensive medical and health service needs [28]. Second, cohabitation with the main caregiver could affect psychological and physical health, which could affect the intent and willingness to use LTC services [29]. Third, the accessibility of supportive care services has been an important consideration in the allocation of long-term care resources in Taiwan. For the elderly who do not live with their children, the transportation distance could affect the early willingness to seek healthcare assistance and the motivation to be discharged. Furthermore, the observed trend in the relationship between CMS level and LTC service usage could be attributed to several factors: (a) greater spousal support and assistance for improving compliance with LTC needs assessments and (b) higher levels of education contribute to making intelligent decisions in selecting the appropriate care. The validity of these assertions is supported by the fact that after adjusting for marital and educational factors, the relationship between CMS and the use of LTC weakened. This indicates that the observed relationship is partly regulated by these mechanisms.

Based on our findings, disability cards and a high body mass index are important predictors of LTC utilization. Based on our research, we found that those with disability cards were more likely to use transportation and respite services, which is consistent with Chen et al. ’s study [16]. Previous studies have reported that excess body weight is associated with higher use and costs of primary care services [30,31]. In addition, about 54% of overweight/obese patients accessed services, using more “personal and professional care” and “transportation” services. A “U”-shaped relationship between weight and disability has been demonstrated in older adults [32], with up to 40% of those with a disability card having a BMI ≥ 24 and 14% having a BMI < 18.5, indicating that the disabilities in our study were dominated by obese and underweight individuals. Whether individuals with a poor BMI use personal care and/or social support services to benefit their health warrants further study. It is necessary to do more research on the health outcomes of older adults with disabilities combined with overweightness and their use of LTC services after discharge.

Our study has some limitations. First, with the lack of a follow-up analysis of subsequent service utilization, we may have underestimated overall service utilization. Having an official platform and being able to share records between institutions should provide better insight into service usage. Second, our study was based on a sample selected from a certain region, which limits its generalizability. The results explain different trends in user utility, suggesting that other factors, in addition to those included in our analysis, could influence the various aspects of the need for LTC. Future research is needed to further explore other determinants. Third, a key contribution of this study is the examination of the seamless integration model regarding LTC 2.0′s needs and planning in the Taiwanese context. However, given the emerging nature of this research focus and the lack of previous studies available, our findings should be interpreted with caution. This study’s results clarify the importance of continuing to plan for the needs of the elderly population in LTC 2.0 with new evidence from Taiwan, which is a rapidly aging East Asian society. Future research should focus on the views of those who do not use services and compare them with those who do use services.

## 5. Conclusions

This study shows that Taiwan provides multiple resources for LTC services. The DP–LTC integrated system has indeed improved service utilization, but there is still an obvious disconnect between the willingness to use and the actual use of the service. This gap emphasizes the importance of communication between users and evaluators so that their needs can be fully reflected in the evaluation results. Strengthening medical teams’ education and training in LTC 2.0 could improve communication. As only the use of transportation services was positively associated with CMS level, the trend in the relationship between CMS level and LTC service usage could be explained by informal care. The impact factors differ based on service type and a relationship between BMI and disability has been confirmed. It was found that people with poor BMI and disabilities used more support services, which is why it is important to consider BMI when allocating resources. This study’s findings provide some insight into the challenges that service providers and policymakers face in implementing DP–LTC care systems.

## Figures and Tables

**Table 1:** Characteristics of the study group (*n* = 2155).

		Frequency	Percent
Age	≤69	433	20.1
	70–79	701	32.5
	≥80	1021	47.4
Gender	Female	1109	51.5
	Male	1046	48.5
Education	none	605	28.1
	primary school	1004	46.6
	junior high and above	490	22.7
Marital status	unmarried	617	28.6
	married	1538	71.4
Income	low income	143	6.6
	general income	2007	93.1
Living area	urban	862	40.0
	rural	1291	59.9
Disability card holder	yes	656	30.4
	no	1499	69.6
Dementia	without	2017	93.6
	with	138	6.4
Body Mass Index	underweight (BMI < 18.5)	255	11.8
	normal (18.5 ≤ BMI < 24)	938	43.5
	overweight(24 ≤ BMI < 27)	473	21.9
	obesity(BMI ≥ 27)	467	21.7
Primary caregiver	foreign caregiver	170	7.9
	daughter	482	22.4
	son	892	41.4
	spouse	508	23.6
	other relatives	87	4.0
	none	16	0.7
CMS level	level 2	43	2.0
	level 3	65	3.0
	level 4	415	19.3
	level 5	428	19.9
	level 6	65	3.0
	level 7	504	23.4
	level 8	635	29.5

**Table 2:** Backward stepwise regression model for factors in referrals to LTC services.

	A. Care and Professional Services			B. Transportation Services			C. Respite Services		
	OR	95% CI	*p* Value	OR	95% CI	*p* Value	OR	95% CI	*p* Value
Female							1.39	(1.02–1.90)	0.036
Married							1.37	(0.96–1.96)	0.087
With disabilities				1.47	(1.09–1.99)	0.012	1.57	(1.15–2.14)	0.005
CMS level (2–8)				1.16	(1.06–1.27)	0.001			
Primary education				1.09	(0.77–1.56)	0.620			
Higher education				1.51	(1.02–2.25)	0.041			
BMI < 18.5 (underweight)	0.87	(0.51–1.48)	0.608	0.69	(0.40–1.19)	0.183			
24 ≤ BMI < 27 (overweight)	1.42	(0.98–2.07)	0.063	1.40	(0.96–2.03)	0.079			
BMI ≥ 27 (obesity)	1.46	(1.01–2.12)	0.045	1.81	(1.27–2.59)	0.001			

OR, odds ratio; CI, confidence interval; CMS, Care Case-Mix System; BMI, body mass index. Variable(s) entered in step 1: age, gender, education, marital status, income, living area, BMI, disability, dementia, main caregiver, and CMS level.

## Data Availability

The data that support the findings of this study are available from Changhua Christian Hospital. Restrictions apply to the availability of these data, which were used under license for this study. Dara are however available from the authors upon request and with permission of Changhua Christian Hospital.

## Abbreviations

ADL: Activities of Daily Living; CCH: Changhua Christian Hospital; CMS: Case-mix system; DP: Discharge planning; IADL: Instrumental Activities of Daily Living; LTC: Long-term care; MDAI: Multi-dimensional Assessment Instrument; SPSS: Statistical Package for the Social Sciences.
